# Gap Junctions Are Essential for Generating the Correlated Spike Activity of Neighboring Retinal Ganglion Cells

**DOI:** 10.1371/journal.pone.0069426

**Published:** 2013-07-23

**Authors:** Béla Völgyi, Feng Pan, David L. Paul, Jack T. Wang, Andrew D. Huberman, Stewart A. Bloomfield

**Affiliations:** 1 Department of Ophthalmology, New York University Langone Medical Center, New York, New York, United States of America; 2 Department of Physiology and Neuroscience, New York University Langone Medical Center, New York, New York, United States of America; 3 Department of Experimental Zoology and Neurobiology, University of Pécs, Pécs, Hungary; 4 János Szentágothai Research Center, Pécs, Hungary; 5 Department of Neurobiology, Harvard Medical School, Boston, Massachusetts, United States of America; 6 Department of Neurobiology, Stanford University School of Medicine, Palo Alto, California, United States of America; 7 Neurosciences Department in the School of Medicine, and Neurobiology Section, Division of Biological Sciences, University of California San Diego, La Jolla, California, United States of America; Universidade Federal do ABC, Brazil

## Abstract

Neurons throughout the brain show spike activity that is temporally correlated to that expressed by their neighbors, yet the generating mechanism(s) remains unclear. In the retina, ganglion cells (GCs) show robust, concerted spiking that shapes the information transmitted to central targets. Here we report the synaptic circuits responsible for generating the different types of concerted spiking of GC neighbors in the mouse retina. The most precise concerted spiking was generated by reciprocal electrical coupling of GC neighbors via gap junctions, whereas indirect electrical coupling to a common cohort of amacrine cells generated the correlated activity with medium precision. In contrast, the correlated spiking with the lowest temporal precision was produced by shared synaptic inputs carrying photoreceptor noise. Overall, our results demonstrate that different synaptic circuits generate the discrete types of GC correlated activity. Moreover, our findings expand our understanding of the roles of gap junctions in the retina, showing that they are essential for generating all forms of concerted GC activity transmitted to central brain targets.

## Introduction

Concerted activity is a common property of cell ensembles in the CNS believed to play a central role in creating a distributed neural code [1 2 3 4]. Correlated spiking serves to convey more information than can be extracted from independent neuron activity [5 6 7 8], which in the visual system includes precise information related to stimulus structure and position [9 10 11 12 13 14]. At central targets, coherent activity supports a number of higher-level brain functions, including perception, attention and feature recognition [15 16 17]. Conversely, spike correlations may be disadvantageous in some cases, reflecting inefficient redundancy of signals inherent to massive interconnectivity of cells, which can limit the coding of information [1 18 19 20]. Such contrasts imply that correlated firing of cell neighbors is derived by a number of different generating mechanisms.

Retinal ganglion cells (GCs) have been a particularly well-studied ensemble, which show a high level of correlated firing. This includes light-independent correlations with varying temporal precision ranging from synchronous activity to relatively loose cross-correlation profiles spanning tens of milliseconds [21 22 23 24 25 26]. Nearby GCs also display coherent activity that is strongly dependent on light stimulus parameters, including intensity, size, contrast, and movement [10 27 28 29]. The finding that correlated spiking can account for over one-half of all GC activity, together with the maintained temporal precision in which retinal signals are propagated to central targets, suggests that correlated activity is vital in encoding visual information [6 15 30 31]. It has been proposed that concerted spiking of GC neighbors provides additional information to the brain, up to 20% more in the primate, thus overcoming the limited bandwidth of the optic nerve [18 24 25]. This idea is supported by the finding that concerted GC activity is enhanced as we move from night to day when additional visual information must be encoded by the retina [29].

While concerted GC activity has an apparent major role in coding visual signals, its generating mechanism(s) remains unclear. Most GCs form gap junctions with GC and/or amacrine cell (AC) neighbors [32 33] and it has been suggested that direct and indirect electrical coupling is responsible for most of the correlated GC activity [6 25 34]. In contrast, some GCs with no apparent coupling show concerted activity as well. Thus it has been posited that shared inputs derived from rod and cone photoreceptor noise provides for the correlated activity of GCs with gap junctional coupling playing little if any role [35 36 37 38]. These contradictory data suggest that the >20 subtypes of retinal GCs may use different strategies to generate activity correlated to that of their neighbors.

To date there has been no comprehensive study of the effects of perturbing chemically-mediated and/or electrically-mediated synaptic transmission to determine the circuits responsible for generating the spontaneous concerted activity of distinct GC subtypes. Therefore, here we used pharmacologic and genetic techniques to differentially manipulate the chemical and electrical synapses in the retina and determine how such intervention changes the concerted activity of the established subtypes of GCs in the mouse retina. Our results demonstrate that a number of discrete circuits, including shared inputs and reciprocal coupling, contribute to the different forms of GC concerted activity. We find, however, that electrical coupling via gap junctions is a constituent of each of these circuits and, moreover, is absolutely essential for the generation of all forms of correlated GC spiking in the mouse retina. These results expand the known roles of gap junctions and electrical coupling in shaping the responses of neuronal ensembles in the retina.

## Results

### Correlated Spiking of Mouse Retinal GCs

**Figure 1:**
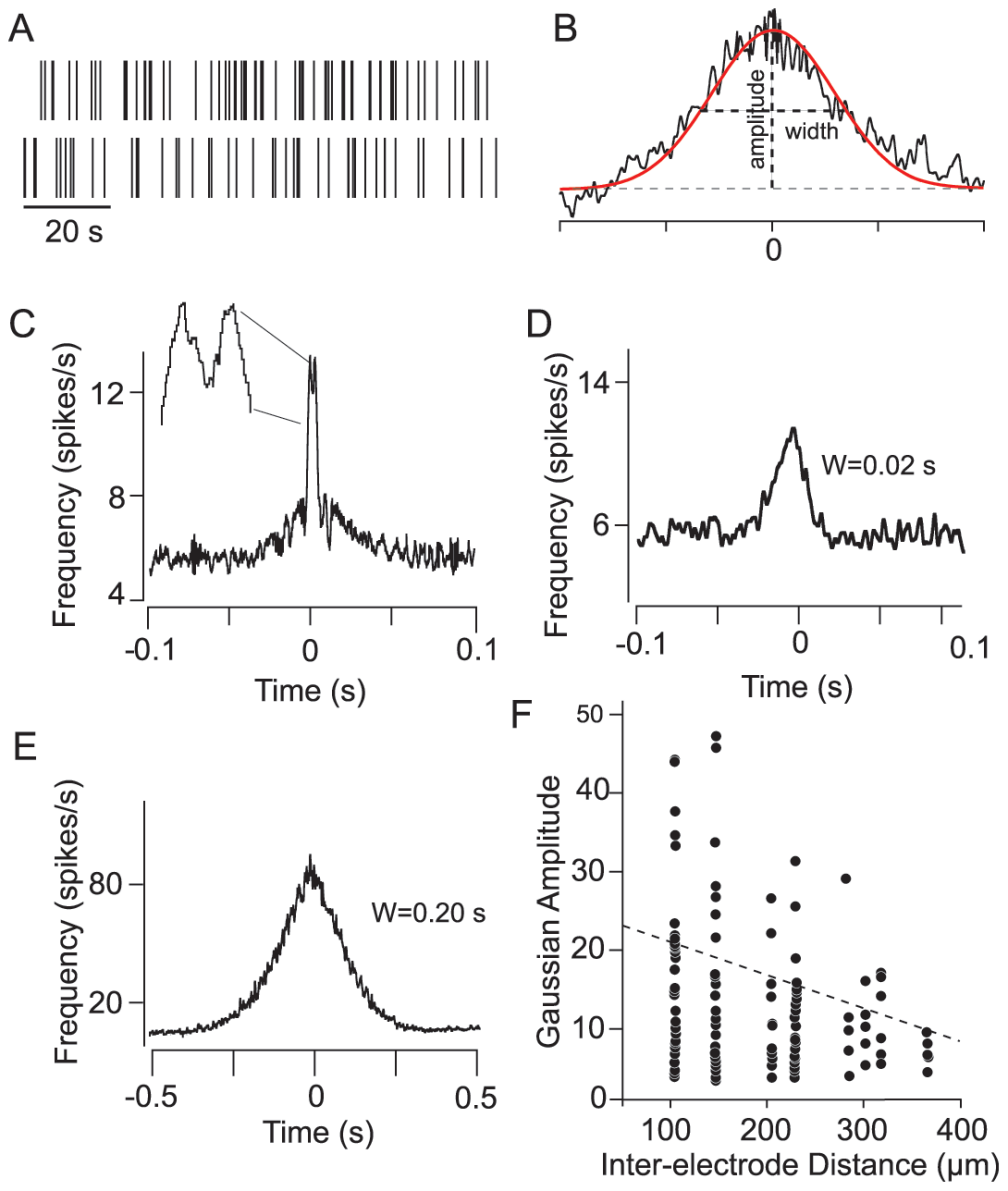
Correlated Spikes of Retinal GCs Occur on Varied Timescales. (A) Panel shows exemplary spontaneous spikes of a GC pair recorded simultaneously. (B) Cross-correlation functions (CCFs) of spontaneous spikes were fit with a Gaussian function from which values for amplitude and width were computed. (C) CCF with a bimodal structure consisting of two peaks separated by a central trough at time zero (inset). Note that the narrow bimodal component is superimposed on a somewhat broader profile. (D and E) CCFs displaying unimodal peaks with different Gaussian widths (W), reflecting differences in the temporal precision of unimodal spike correlations. (F) Scatterplot demonstrating an inverse relationship between the Gaussian amplitudes for unimodal CCFs and the inter-somatic distance of the recorded GC pairs. The dashed line indicates a linear regression fit of the data.

To initially determine the synchronous patterns of activity in the mouse retina, spontaneous spike trains were recorded from mouse GCs either with paired tungsten electrodes (PTE; n = 364) or with a multi-electrode array (MEA) consisting of 60 electrodes with a regular spacing of 100 µm (n = 5108) **(** **Figure 1A** **)**. The PTE recordings were made under visual guidance in order to randomize sampling of the >20 different subtypes of GCs found in the mouse retina [33]. In addition, PTE recordings were usually made from GC somata with similar size and shape to increase the likelihood that GCs of the same subtype were paired. In contrast, the MEA recordings, which were made blindly, necessarily sampled activity from a totally random cohort of GC subtypes. The two recording methods were complementary in terms of the inter-somatic distances of GCs recorded simultaneously in that the PTE recordings were made from visualized somata separated by 15–200 µm as limited by the microscopic field-of-view, whereas the MEA inter-electrode spacing translated to recorded GC inter-somatic distances of 100–1000 µm.

To analyze the concerted spontaneous spike activity between GCs we computed CCFs for GC pair-wise recordings, which were then fit with a Gaussian function from which the width (W) and amplitude (A) parameters were measured **(** **Figure 1B** **)**. Based on the shape of the CCFs and their Gaussian parameters, we were able to differentiate them into two groups. One CCF type displayed a bimodal profile with fast rising and narrow (W = 1.50±0.19 ms) peaks and with amplitudes that were surprisingly consistent across the sample population (A = 13.3±1.7 spikes/s) **(** **Figure 1C** **)**. The bimodal peaks were separated by a central trough and usually displaced symmetrically from time zero with similar latencies of 1.10±0.08 ms. The bimodal peaks were often superimposed on broader profiles in the CCF **(** **Figures 1C** **and** **5B,C** **)**.

Interestingly, the GC pairs showing bimodal CCF components were encountered very rarely. They comprised only 7.4% of the total PTE paired recordings (n = 27) and only 0.3% of the paired MEA recordings (n = 16). We found that bimodal CCFs were only displayed by GC pairs whose somata were separated by ≤100 µm. This likely explains why they were seen so infrequently, particularly for recordings using the MEA array in which 100 µm was the minimal distance between GCs due to electrode spacing.

In addition to the bimodal CCFs, we also encountered paired recordings that generated CCFs with a single peak centered at time zero indicating a high degree of synchrony (PTE, n = 135, 37.1% of all pairs; MEA, n = 151, 3.0% of all pairs). These unimodal CCFs showed considerable variability in both their widths (4–400 ms) and amplitudes (5–50 spikes/s) **(** **Figure 1D,E,F**). We found that as the inter-electrode distance increased, resulting in recordings from GC pairs with wider separation, the amplitude of the CCF decreased **(** **Figure 1F** **)**. These results indicate that both the strength and the temporal precision of spike synchrony between GC pairs declined as inter-somatic distances increased.

It is important to note that of the >5,000 paired recordings made, nearly 50% of PTE recordings showed statistically significant spike correlations (99% confidence above chance), yet only 3% of the MEA recordings showed significant correlations. As noted, spike correlations were not seen for GC pairs separated by >400 µm, and so the higher yield found for PTE recordings would appear to reflect the fact that the MEA technique often sampled GCs that were relatively distant. However, we found that over one-half of the MEA paired recordings without statistically significant spike correlations were from GCs separated by <400 µm. This argues that the inter-somatic distance was not the principle factor responsible for the relatively low number of cell pairs found with correlated activity. Instead, the greater number of PTE-recorded GC spike correlations could reflect the greater chance of obtaining pair-wise recordings from the same subtype of GCs.

### Effects of Pharmacological Blockade on Spike Correlations

**Figure 2:**
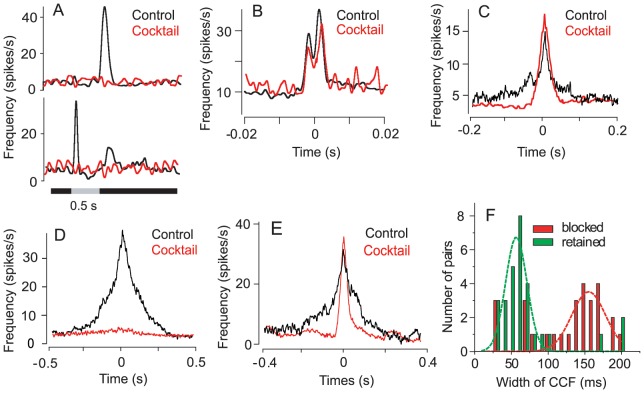
Blockade of Chemical Synaptic Activity Abolishes Broad Unimodal CCFs, but Not Medium Unimodal or Bimodal CCFs. (A) A cocktail of neurotransmitter antagonists effectively eliminate the light-evoked responses of both ON (bottom) and OFF (top) GCs. Presentation of the light stimulus (I = 3000 R*/rod/sec) is indicated by the grey bar. (B and C) Application of the cocktail does not significantly alter spike correlations reflected in bimodal CCFs or unimodal CCFs with relatively small (medium) widths. (D) In contrast, spike correlations reflected in unimodal CCFs with relatively broad widths are largely eliminated when chemical synaptic activity is blocked. (E) Example of a unimodal CCF in which application of the cocktail of antagonists abolished the broad profile revealing a surviving narrower component of medium width. (F) Summary of the effects of cocktail on spike correlations reflected in unimodal CCFs. The correlations indicated by broad CCFs are readily blocked by cocktail treatment, whereas those reflected by more narrow unimodal CCFs are largely retained.

To determine the retinal circuits responsible for generating the two types of GC correlated activity, we employed a combination of pharmacologic and genetic approaches to selectively ablate chemical and/or electrical synapses. In initial experiments, we applied a cocktail of transmitter antagonists to block all excitatory and inhibitory chemical synapses (see Methods). As expected, application of the cocktail blocked signals derived from the photoreceptors and thereby eliminated the light-evoked responses of all ON and OFF GCs we sampled **(** **Figure 2A** **)**.

However, we found that blockade of chemical transmission had no significant effect on bimodal CCFs of spontaneous spiking (n = 10), which, again, were found only for closely spaced GC pairs **(** **Figure 2B** **)**. In contrast, blockade of chemical synaptic transmission had differential effects on the unimodal CCFs. Whereas application of the cocktail of blockers largely eliminated the relatively broad unimodal CCFs (W>100 ms; n = 62), the more narrow unimodal CCFs (n = 23) were largely unaffected **(** **Figure 2C,D** **)**. For some GC pairs that were separated by ∼100 µm (n = 19/31), we found that ablation of the broad correlation with the chemical synaptic blockers often revealed a more narrow correlation profile in the CCF **(** **Figure 2E** **)**. This indicates that the CCFs of some GC pairs reflected a superposition of multiple profiles with different widths. However, we found that correlations of GCs separated by 200–400 µm were almost always presented as broad CCFs with W >100 ms (i.e., bimodal or more narrow unimodal profiles were rarely seen) and were consistently abolished by blockade of synaptic transmission (n = 25) **(** **Figure 2F** **)**. Taken together, these results indicated that spontaneous spike correlations of well-separated GCs, as reflected by broad CCFs, were dependent on functional chemical transmission.

Based on the differential action of the cocktail on spike correlations, we refer below to unimodal CCFs with W >100 ms as *broad* and those with W <100 ms as *medium*. Following this nomenclature, we will term all bimodal CCF profiles as *narrow*, given that the Gaussian width of each peak was ∼2 ms. This terminology is consistent with that used previously to describe GC correlations in a number of species [10 23 25 34 35 38].

**Figure 3:**
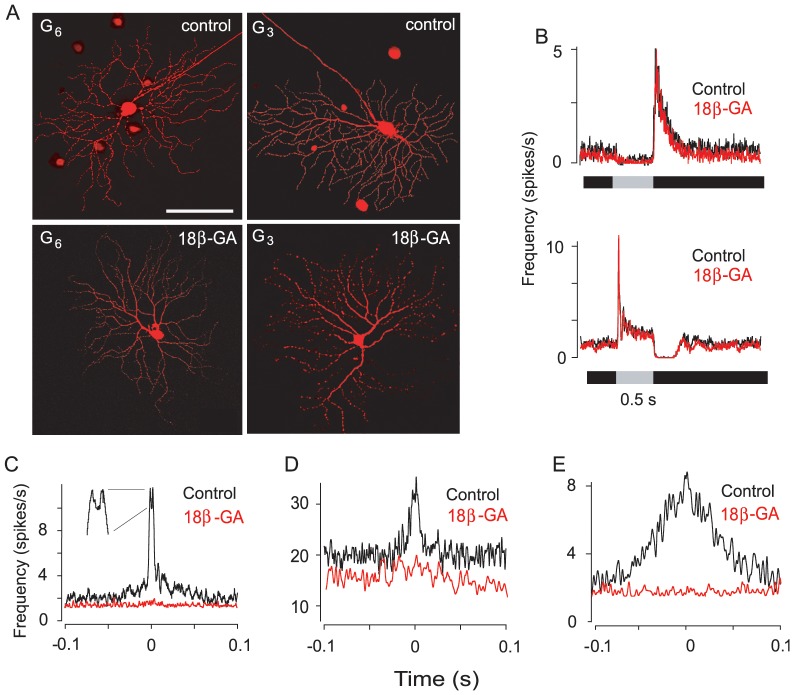
Blockade of Gap Junctions Eliminates All Types of GC Spike Correlations. (A) Application of 18β-GA (25 µM) effectively blocks the Neurobiotin tracer coupling of GCs. Micrographs show elimination of tracer coupling of an ON G_6_ cell, which shows coupling to ACs under control conditions, and an OFF G_3_ cell, which shows coupling to GC and AC neighbors under control conditions. Scale bar = 100 µm. (B) The blockade of gap junctions with 18β-GA does not significantly alter the full-field light responses of ON (bottom) and OFF (top) GCs. Presentation of the light stimulus (I = 3000 R*/rod/sec) is indicated by the grey bar. (C,D, and E) Blockade of gap junctions effectively eliminated all spike correlations reflected in narrow, medium, and broad CCFs.

The relative insensitivity of medium and narrow spike correlations to the cocktail of blockers indicated that chemical synapses do not play a critical role in generating GC spike correlations with short time scales. To determine whether electrical synaptic transmission played a role, we applied the gap junction blocker 18-beta-glycyrrhetinic acid (18β-GA). Application of 18β-GA at concentrations >30 µM has been shown to affect sodium currents in expression and arterial systems [65 66 67]. However, at the relatively low concentration of 25 µM, which we employed, 18β-GA effectively uncoupled cells **(** **Figure 3A** **)** and had no significant effect on the photopic, light-evoked responses of GCs (n = 91) **(** **Figure 3B** **)**, arguing against any non-specific effect on cell excitability. Moreover, application of 18β-GA effectively eliminated the correlation of spontaneous spiking of GC pairs as reflected in the loss of both narrow (n = 8) and medium (n = 12) CCF profiles **(** **Figure 3C,D** **)**. Interestingly, we found that 18β-GA also abolished the correlated spiking reflected in broad CCFs (n = 4) **(** **Figure 3E** **)**. Taken together with our earlier results, these data indicate that electrical coupling via gap junctions, rather than chemical synaptic circuits, mediates correlation of spontaneous GC spiking with high temporal precision as reflected in the narrow and medium CCFs. These data further indicate that broad correlations are dependent on both chemical and electrical synaptic activity.

### Broad CCFs are Absent in the Cx36 KO Mouse Retina

**Figure 4:**
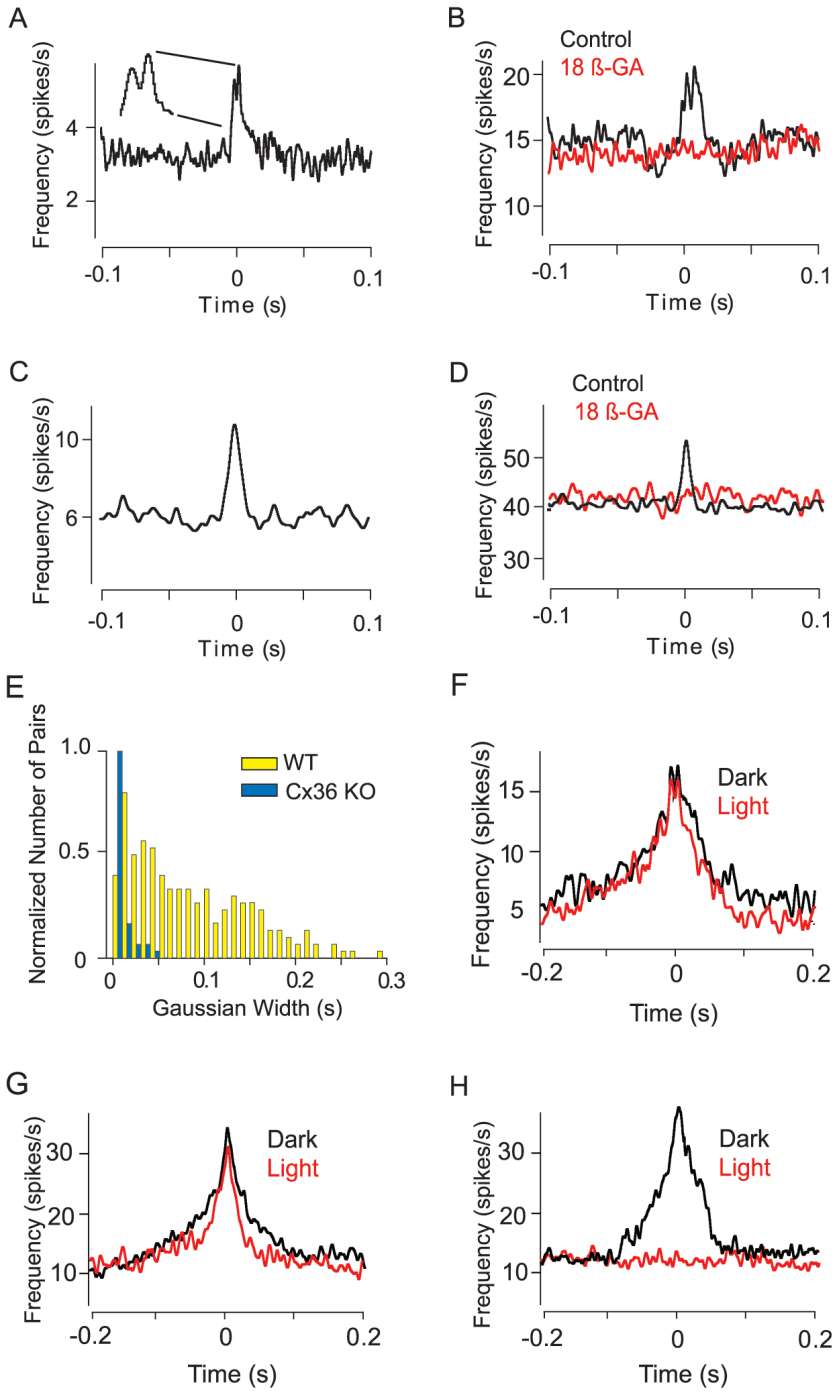
Broad Spike Correlations are Absent in the Cx36 KO Mouse Retina. (A–D) GC pairs in the Cx36 KO mouse retina displayed either narrow bimodal (A) or medium (C) unimodal spike correlations. Both narrow bimodal and medium spike correlations were abolished following application of 25 µM 18β-GA (B and D). (E) Bar graph summarizing changes in the relative frequency of unimodal spike correlations when Cx36-expressing gap junctions are ablated in the KO mouse retina. (F–H) Light adaptation of the WT mouse retina with a photopic (I = 3000 R*/rod/sec) background stimulus does not alter narrow (F) or medium (G) spike correlations, but eliminates the broad (H) correlations.

As a second method to study the effect of gap junction ablation on spike correlations, we measured CCFs in the Cx36 knockout (KO) mouse retina using both the PTE (n = 168) and the MEA (n = 5989) recording methods. Overall, we found that correlation of spontaneous spikes in GC pairs were encountered even less frequently in Cx36 KO mouse retinas (n = 96; 1.5%) than in the WT (n = 392; 3.7%). Whereas we showed that spike correlations corresponding to both the narrow and medium CCFs were abolished by blockade of gap junctions with 18β-GA, these types of spike correlations were still encountered in the Cx36 KO mouse retina **(** **Figure 4A,C,E** **)**. However, these types of CCFs, particularly the medium correlations, were encountered far less frequently in the KO retina than in the WT **(** **Figure 4E** **)**.

In contrast, there was a complete absence of the broad subtype of CCF in the KO, resulting in a dramatic decrease in the number of unimodal spike correlations. Overall, the CCFs in Cx36 KO retinas showed greater temporal precision than those in the WT suggesting that this surviving cohort were dependent on gap junctions expressing a connexin type different from Cx36 **(** **Figure 4E** **)**.

Overall, our results suggested that the spike correlations of GC pairs showing broad CCFs, as well as many medium CCFs, were dependent on gap junctions that express Cx36, whereas narrow correlations were not. To confirm that the remaining narrow and medium spike correlations seen for GC pairs in the Cx36 KO were still dependent on electrical coupling, we tested whether they were sensitive to the addition of the non-specific gap junction blocker 18β-GA. As we found in the WT, addition of 18β-GA effectively eliminated all narrow (n = 5) and medium correlations (n = 9) tested in the Cx36 KO retina **(** **Figure 4B,D** **)**.

Previous studies reported that broad correlations between GCs in dark-adapted cat and primate retinas, similar to what we report here in the mouse, were largely absent under light-adapted conditions suggesting that they were derived from rod-driven pathways [22 37]. Similarly, we found that light adaptation of WT mouse retina (background light: λ = 528 nm; I = 3000 Rh*/rod/s) had no effect on either narrow bimodal (n = 12/12) or medium unimodal CCFs (n = 23/23), but abolished nearly all broad CCFs tested (n = 12/14) **(** **Figure 4F,G,H** **)**. These results indicate that narrow and medium GC spike correlations are unaffected by the adaptation state of the retina, whereas the broad correlations are derived from scotopic signals carried by the rod pathways.

### OFF α-GC Neighbors Show Spike Synchrony on Multiple Time Scales

Our finding that narrow and medium CCFs were dependent on electrical coupling, but survived blockade of chemical synaptic transmission, suggested that the gap junctions involved are located proximally in the inner retina, likely expressed directly by GCs. The inner retina includes a wide variety of gap junctional coupling patterns, principally GC-to-GC and GC-to-AC junctions (33). To determine the relationship between these different coupling patterns to the different types of spike correlations, we next targeted and performed pair-wise recordings from identified GC subtypes.

**Figure 5:**
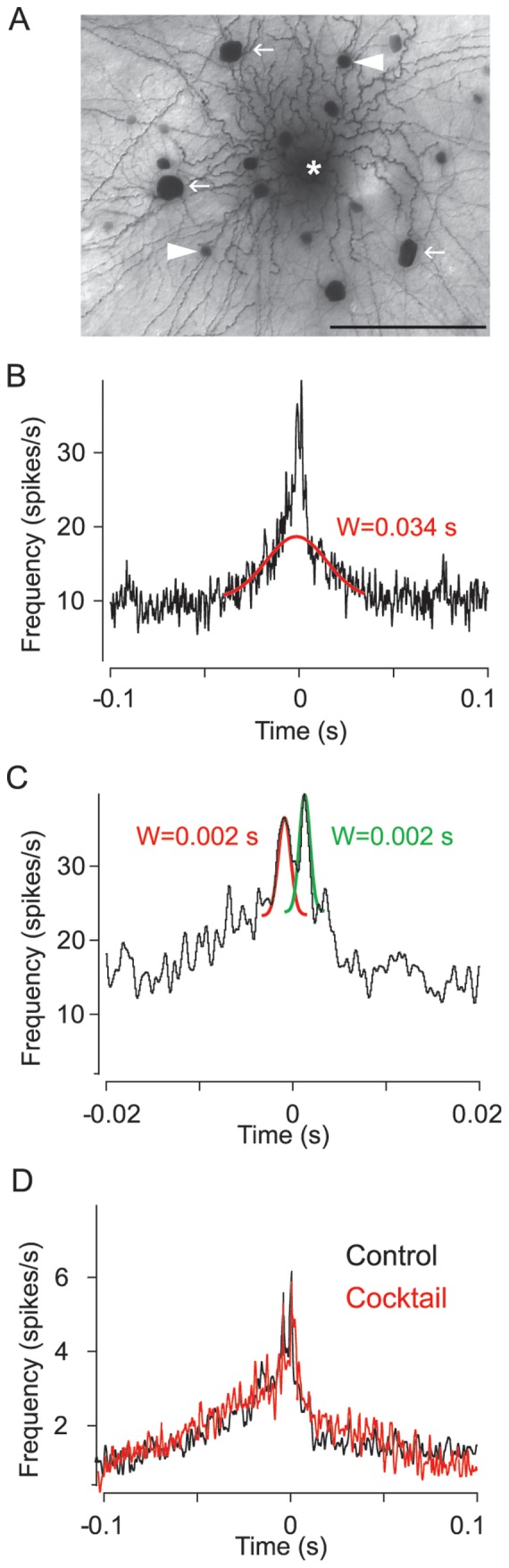
Neighboring OFF α-GCs Exhibit Both Narrow and Medium Spike Correlations. (A) Tracer coupling pattern revealed after iontophoresis of Neurobiotin into a single OFF α-GC (asterisk). The OFF α-GC is coupled to an array of neighboring α-GCs (white arrows) and a cohort of nearby ACs (white arrowheads). Scale bar = 100 µm. (B and C) The CCF generated for the spontaneous spike activity of a pair of OFF α-GCs exhibits a characteristic superposition of two components: a unimodal component with medium Gaussian width (B) upon which a narrow, bimodal component (C) is superimposed. (D) Neither narrow nor medium spike correlations of OFF α-GC pairs were eliminated by the blockade of chemical synaptic activity.

In initial experiments, we targeted the OFF α-GCs in the WT mouse retina, which have previously been shown to maintain gap junctional coupling to OFF α-GC neighbors and at least two subpopulations of ACs [33 39 40]. We used infrared (IR) guidance to target mouse OFF α-GCs to maintain dark-adapted conditions. The OFF α-GCs exhibited large spherical or somewhat elongated cell bodies with eccentric nuclei and were thereby easily targeted. Subsequent Neurobiotin injections confirmed the identity of OFF α-GCs based on well-established soma/dendritic morphological criteria and their tracer coupling patterns [33 41] **(** **Figure 5A** **and** **7A,B** **)**.

Casual observation of the spontaneous impulse activity of paired OFF α-GC recordings indicated the presence of many individual spikes and spike bursts of one cell that were time-locked to the activity of its neighbor. This likely reflected the fact that a spike in one cell could induce activity in its coupled neighbor. The CCFs generated for OFF α-GC paired recordings (n = 27) displayed robust spike synchronization with a complex structure **(** **Figure 5B** **)**. Most apparent was a bimodal profile, consisting of individual peaks with Gaussian widths of 1.9±0.2 ms that were symmetrically displaced from the prominent trough by 1.3±0.1 ms corresponding to narrow correlations **(** **Figure 5C** **)**. These narrow CCFs in the wild type mouse were always superimposed on a unimodal profile with a Gaussian width of 40.0±5.1 ms, consistent with the time scale of medium spike correlations **(** **Figure 5B** **)**. Consistent with our earlier findings, the bimodal and unimodal peaks of OFF α-GC pair recordings were eliminated by a blockade of electrical synaptic transmission (n = 4), but were unaffected by the blockade of chemical transmission (n = 4) or light adaptation (n = 6) **(** **Figure 5D** **)**.

### Narrow and Medium Spike Correlations are Mediated by GC-to-GC and GC-to-AC Coupling, Respectively

While our results showed that neighboring OFF α-GCs exhibit both narrow and medium correlated spike activity, it remained unclear whether these were generated separately by GC-to-GC or GC-to-AC coupling, both which were expressed by these cells. To illuminate the relationship between the pattern of coupling and spike correlation, our strategy was to delete one type of coupling and determine how this affected narrow and medium CCFs. The Cx36 KO mouse retina appeared to be an appropriate model in that it was reported that OFF α-GC-to-AC gap junctions were ablated leaving coupling only between neighboring OFF α-GCs [39 40]. However, Schubert et al. [42] found that coupling to both neighboring OFF α-GCs and ACs was abolished in the Cx36 KO mouse retina, suggesting that Cx36 was localized to both sets of gap junctions. Although, this discrepancy could reflect differences in the two Cx36 KO mouse lines, it does question the validity of our Cx36 KO mouse strain as a suitable model for our studies.

**Figure 6:**
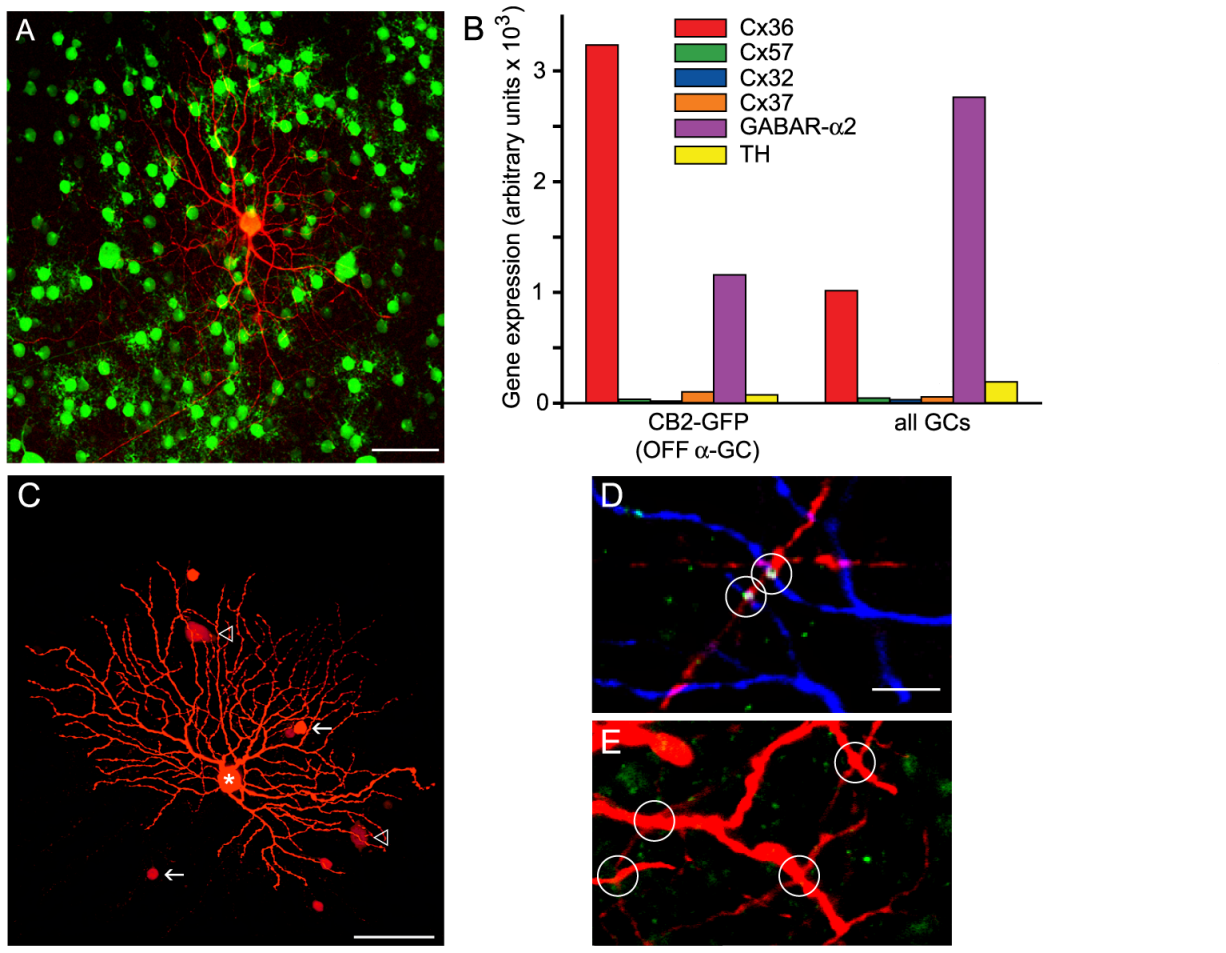
OFF α-GCs Express Cx36 In AC-To-GC, but Not GC-To-GC, Gap Junctions. (A) Example of the morphology of a single Neurobiotin injected GFP-positive GC (red) in the CB2-GFP retina corresponding to that of an OFF α*-*GC (41). Scale bar = 50 µm. (B) Relative gene expression of Cx36, Cx57, Cx32, Cx37, GABA receptor α2 subunit (GABAR- α2) and tyrosine hydroxylase (TH) in CB2+ OFF α-GCs. (C) A Neurobiotin-injected OFF *α-*GC in the WT mouse retina displaying the characteristic tracer coupling to neighboring GCs and ACs. Scale bar = 50 µm. (D) Single optical section (thickness = 0.5 µm) of a confocal image showing the colocalization of Cx36 immunolabeled puncta (green) with dendritic crossings of Neurobiotin-injected OFF α*-*GC (blue) and tracer coupled ACs (red). Scale bar = 10 µm. (E) No significant colocalization of Cx36 puncta (green) are found at dendritic crossings of Neurobiotin coupled neighboring OFF α*-*GC dendrites (red).

To clarify this discrepancy, we examined the expression of Cx36 in OFF α-GC gap junctions at the transcriptional and structural levels. To efficiently target OFF α-GCs we exploited the CB2-GFP mouse line in which the coding sequence of green fluorescent protein (GFP) is genetically linked to the expression of calretinin [41]. In this animal, GFP is selectively expressed by OFF α-GCs and AII amacrine cells with somata located in the GCL and the INL, respectively **(** **Figure 6A** **)**. We harvested CB2-GFP positive GCs (OFF α-GCs) with a combination of imunopanning procedure and fluorescence-activated cell sorting (FACS: see Methods) and assayed transcript expression levels by measuring signal strength of hybridized cRNA fragments **(** **Figure 6B** **)**. We found that the Cx36 mRNA level was three times greater in OFF α-GCs (3235; expression levels are given as arbitrary units) than in the general GC population (1015). In addition, the Cx36 mRNA level of OFF α-GCs was somewhat higher than the positive control GABA_R_-α2 subunit level and 10-1000 times higher than levels of the negative controls, including Cx57, Cx32, Cx37, and tyrosine-hydroxylase (TH) (see Methods). Overall, these results indicated that OFF α-GCs express Cx36 mRNA transcripts.

In the next set of experiments, we combined Neurobiotin labeling of OFF α-GCs with immunolabeling of Cx36 in retinal tissue. We confirmed that OFF α-GCs are tracer coupled to neighboring OFF α-GCs and cohorts of ACs **(** **Figure 6C** **)**. Moreover, we found that immunolabeled Cx36 puncta were present at many dendritic crossings established by OFF α-GCs and the tracer-coupled ACs **(** **Figure 6D** **)**. In contrast, no obvious Cx36 punctate label was detected at dendritic crossings between neighboring OFF α-GCs dendrites **(** **Figure 6E** **).** These data strongly suggest that OFF α-GCs express at least two different connexin subunits, Cx36 in the gap junctions made with AC neighbors and a yet unidentified connexin(s) in the homologous gap junctions between OFF α-GC neighbors. Taken together, these data confirmed that the coupled OFF α-GC network is an appropriate model to study the contributions of GC-to-GC and GC-to-AC coupling to the different patterns of concerted spike activity.

**Figure 7:**
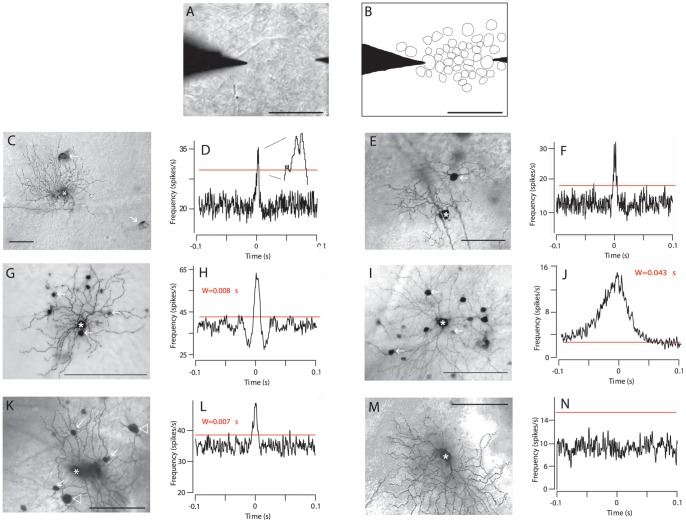
GC-to-GC Coupling Underlies Narrow Spike Synchrony, Whereas AC-To-GC Coupling Mediates the Medium Spike Correlations. (A) Video image of a PTE recording from a pair of OFF α-GCs in the living mouse retina. Scale bar = 50 µm. (B) Schematic of the image in panel *A* showing how the mosaic of somata in the GCL was outlined to determine the identity of the GCs recorded in pair wise recordings in which one cell was injected with Neurobiotin. (C) Neurobiotin-injected OFF α-GC in the Cx36 KO mouse retina shows only homologous coupling to OFF α-GC (arrows). Scale bar = 100. (D) Spontaneous spiking of a pair of OFF α*-*GCs in the Cx36 KO mouse retina produces a CCF with only a narrow, bimodal component. Red line indicates 99% confidence level. (E) A Neurobiotin-injected G_17_ GC (asterisk) shows coupling to neighbor G_17_ GCs (arrow). Scale bar = 100 µm. (F) Spike correlations of G_17_ GC pairs are reflected by narrow, bimodal CCF components. bimodal CCFs. (G) Neurobiotin-injected G_6_ cell (asterisk) shows the characteristic tracer coupling to neighboring ACs (arrows). Scale bar = 150 µm. (H) Correlated spikes of a pair of G_6_ cell neighbors are reflected by a CCF with a unimodal profile with medium Gaussian width. (I) Neurobiotin-injected G_1_ cell (asterisk) in the WT mouse retina shows characteristic tracer coupling to multiple cohorts of neighboring ACs (arrows). Scale bar = 150 µm. (J) Correlated spikes of a pair of G_1_ cell neighbors in the WT are reflected by a CCF with a unimodal profile with medium Gaussian width. (K) Neurobiotin-injected G_7_ cell (asterisk) in the WT mouse retina shows characteristic tracer coupling to neighboring GCs (arrowheads) and ACs (arrows) (left). Scale bar = 150 µm. (L) Correlated spikes of a pair of G_7_ cell neighbors in the WT are reflected by a CCF with medium unimodal profile. (M) Neurobiotin-injected G_7_ cell (asterisk) in the Cx36 KO retina shows no tracer-coupling. Scale bar = 150 µm. (N) No significant spike correlations between G_7_ cell neighbors in the Cx36 KO retina are reflected by a CCF with a flat profile.

To record from pairs of GCs of the same subtype, we visualized and targeted somata of similar size and shape **(** **Figure 7A** **)**. This was confirmed by injecting one GC with Neurobiotin and identifying the other by comparing the mosaic of cells in the GCL in the living retina to that after histologically processing (**Figure 7B**, see Methods).

As we reported previously [33], OFF α-GCs in the Cx36 KO retina are tracer coupled only to OFF α-GCs neighbors as coupling to neighboring ACs is abolished **(** **Figure 7C** **)**. We therefore recorded from pairs of OFF α-GCs in the KO mouse to determine whether deletion of GC-to-AC gap junctions in the Cx36 KO mouse retina affected the correlated activity between OFF α-GCs neighbors. Indeed, we found that neighboring OFF α-GC pairs (n = 22) in the Cx36 KO showed narrow bimodal correlated spike activity consisting of two peaks with symmetrical latencies of 1.1±0.1 ms from a trough at time zero, a Gaussian width of 2.4±0.3 ms, and a Gaussian amplitude of 14.0±1.8 spikes/s **(** **Figure 7D** **)**. The Gaussian parameters for the narrow CCFs of OFF α-GC pairs in the KO mouse were not statistically different from those measured for narrow CCFs in the WT counterparts (*p*>0.1 for all parameters on paired *t*-test). Importantly, we found no medium profiles in the CCFs of OFF α-GCs in the Cx36 KO mouse retina, a clear difference from that seen in the WT retina. Overall, these findings suggested that direct coupling between neighboring GCs generates narrow spike correlations.

We next performed pair-wise recordings from different subtypes of GCs that showed different patterns of coupling [33] to establish the temporal properties of their spontaneous, concerted activity. Recordings from G_17_ GCs in WT mouse retina (ON-OFF DS or type 2) [43], which express homologous coupling **(** **Figure 7E** **)**, showed spike correlations on a narrow time-scale, similar that found for OFF α-GCs in the Cx36 KO retina **(** **Figure 7F** **)**. In contrast, we found that paired recordings from neighboring G_6_ GCs in the WT mouse retina, which are tracer coupled indirectly via two populations of displaced ACs within the GCL [33], showed correlated spikes in the form of medium CCFs with a unimodal peak at time zero **(** **Figure 7G,H** **)**. A second GC subtype coupled indirectly via ACs is the G_1_ cell, which is one of the largest GCs in the mouse retina. The G_1_ cell is coupled to two subtypes of neighboring ACs with the larger subtype identified as a polyaxonal cell [33] **(** **Figure 7I** **)**. Recordings from pairs of neighboring G_1_ cells showed correlated spike activity represented as unimodal CCFs with medium width **(** **Figure 7J** **)**. In the WT mouse retina, the G_7_ cells show coupling to both GCs and ACs, yet paired recordings from this subtype showed only medium unimodal spike correlations (n = 1; **Figure 7K,L**). However, in contrast to OFF α-GCs, which maintained GC-to-GC coupling and narrow spike correlations in retinas where Cx36 was ablated, all tracer coupling of G_7_ GCs was abolished in the Cx36 KO mouse retina **(** **Figure 7M** **)** and all significant spike correlations were lost **(** **Figure 7N** **)**. We interpret this to likely reflect that G_7_ GCs are not coupled directly in the WT retina and that the apparent homologous coupling actually arises indirectly via a GC-to-AC-to-GC route.

In total, we obtained paired recordings from 10 different GC subtypes in both the WT and Cx36 KO mouse retinas **(** **Table 1** **)**. We found that cells showing GC-to-GC coupling displayed narrow bimodal CCFs, whereas cells with GC-to-AC coupling showed medium unimodal CCFs. Consistent with these, we also found that GC subtypes coupled to both GC and AC neighbors, with the noted exception of G_7_ cells, often displayed superimposed narrow and medium CCF profiles. Finally, pair-wise recordings from GCs that were not tracer coupled did not showed spontaneous spike correlations that were statistically above chance (n = 4).

**Table 1:** Coupling Pattern and Spike Correlations of GC Subtypes.

Cell Pair Subtype	Coupling	CCF
G_3_ (n = 8); WT	GC; AC	Narrow bimodal; medium unimodal
G_7_ (n = 1); WT	GC; AC	Narrow bimodal; medium unimodal
G_6_ (n = 3); WT	AC	Medium unimodal
G_1_ (n = 1); WT	AC	Medium unimodal
G_2_ (n = 5); Cx36 KO	AC	Medium unimodal
G_17_ (n = 4); Cx36 KO	GC	Narrow bimodal
G_3_ (n = 9); Cx36 KO	GC	Narrow bimodal
G_6_ (n = 1); Cx36 KO	No coupling	No correlations
G_21_ (n = 1); Cx36 KO	No coupling	No correlations
G_7_ (n = 2); Cx36 KO	No coupling	No correlations

## Discussion

There are two basic circuits by which correlated activity between neighboring neurons can be achieved: shared synaptic inputs derived from a common presynaptic source and reciprocal interactions via gap junction-mediated electrical transmission. Both circuits exist for retinal GCs, which include shared bipolar cell inputs carrying light-independent photoreceptor noise and extensive electrical coupling. In this study, we used pharmacologic and genetic manipulations to differentiate the contributions of these two circuits to the robust concerted, spontaneous spike activity of GCs. In addition, we directly examined the correlated activity of specific GC subtypes with defined coupling patterns. We draw several general conclusions from our data. First, we found that different synaptic circuits, subserved by chemical and/or electrical synapses, are responsible for the different forms of concerted GC activity distinguished by their temporal properties. Second, shared inputs presumably derived from photoreceptor noise contributed only to correlations with the weakest temporal precision, although this accounted for about 20% of GC correlated activity. Third, functional gap junctions and electrical coupling were absolutely essential for the generation of all concerted activity of GCs in the mouse retina. Below, we consider these findings in detail and their implications for the role of concerted GC activity in visual signaling.

### Different Synaptic Circuits are Responsible for the Three Types of Concerted GC Spiking

Two fundamental types of correlations of spontaneous spiking were found for GC pairs in the mouse retina, one with prominent bimodal peaks separated by 1-3 ms and a second with a unimodal profile indicative of synchronous spike activity. The bimodal CCFs computed for mouse GCs are similar to the narrow spike correlations previously described in many vertebrate species, including cat [23], salamander [25], rabbit [26 34], and primate [35]. Based on its relatively fast kinetics, it was speculated that the narrow, bimodal CCF is a signature for reciprocal interactions between GCs coupled via gap junctions [23 25 26 35]. Consistent with this idea, we found that narrow, bimodal correlations were insensitive to blockade of chemical synaptic transmission, but were readily eliminated by application of gap junction blockers. An additional question is whether the bimodal CCFs reflect direct electrical coupling between GC neighbors and/or indirect coupling via intermediary ACs [23 25 26 34 35 44 45]. The present results indicate that direct coupling between GCs underlies the narrow correlations. First, we found that only neighboring GCs with somata separated by ≤100 µm and thus capable of dendritic overlap showed bimodal correlations, consistent with a previous report in the rat [46]. Second, only those identified GC subtypes that displayed direct coupling also maintained narrow, bimodal correlations **(** **Table 1** **)**. Third, GC subtypes that showed both direct and indirect coupling in the WT animal, but displayed only direct coupling in the Cx36 KO mouse retina, maintained only the narrow spike correlations following connexin deletion.

A second cohort of spike correlations were represented by unimodal CCFs with peak widths ranging from 4 to 400 ms, similar to light-independent correlated firing reported in a number of species [21 22 25 26 35]. We found that unimodal correlations with higher temporal precision (medium) were insensitive to chemical blockade, whereas those represented by broader CCFs with widths >100 ms were eliminated by application of a cocktail of chemical synaptic blockers. In contrast, the medium correlations were largely eliminated by pharmacologic blockade of gap junctions or genetic ablation of connexins. Taken together, these data indicate that electrical synaptic circuits are critical elements in the generation of medium correlations. Specifically, medium CCFs were found exclusively for GC subtypes coupled indirectly via intermediary ACs. The OFF α-GCs in the Cx36 KO mouse retina where indirect coupling was eliminated, also showed a loss of medium, unimodal spike correlation. We therefore conclude that indirect electrical coupling via ACs is essential for generating medium spike correlations between GC pairs in the mouse retina. The generating circuits for the temporally precise narrow and medium spike correlation demonstrated here for the mouse retina parallel those proposed for GCs in the salamander [25], suggesting that the generating circuitry is stereotypic and common across diverse species.

It has been suggested that GC correlations with the broadest temporal properties are produced by shared chemical synaptic inputs, likely from bipolar cells carrying light-independent rod photoreceptor noise [22 35 37]. The present findings provide additional support for this idea. First, we found that GCs with the greatest inter-somatic distances, where dendritic overlap necessary for electrical coupling was minimized, displayed the broadest CCFs. Second, the broad spike correlations were abolished when chemical synaptic transmission was blocked pharmacologically. Third, the broad correlations found in dark-adapted retinas were lost following light adaptation resulting in rod photoreceptor saturation. Together, these data suggest that the broad correlations arise from shared inputs to GCs carrying light-independent noise originating in the rod photoreceptors. However, we found that the broad correlations were critically dependent on functional gap junctions, particularly those expressing Cx36. These apparently incongruous data may be explained by the fact that Cx36-expressing gap junctions are essential elements to the different rod pathways in the mammalian retina. Indeed, ablation of Cx36 completely blocks the secondary rod pathways and seriously impairs transmission across the primary rod pathway [47 48 49].

### Species Comparison

Overall, our results establish conclusively for the first time that stereotypic synaptic circuits, particularly electrical coupling, are essential for generating the different GC spike correlations segregated by their temporal precision. Our results are largely consistent with and extend findings in the cat, rabbit, rat, salamander, and primate retinas [21 22 23 25 26 34 35 37 46]. However, physiological and computational modeling studies in the primate have suggested that while electrical coupling generates the narrow bimodal CCFs, they play only a minor role in the overall correlated GC activity. Instead, it has been posited that shared rod and cone photoreceptor noise is the dominant mechanism responsible for GC concerted activity, particularly medium correlations, in the primate retina [35 37 38 50]. This discrepancy with the present results may reflect species differences in at least some of the mechanisms generating the different forms of GC correlations. However, many of the circuit manipulations performed here in the mouse (e.g., gap junction blockade) have not been carried out as yet in the primate and need to be performed to corroborate results based on mathematical models of the physiological data. Moreover, as pointed out below, shared inputs and reciprocal electrical coupling need not be viewed as mutually exclusive mechanisms as signals derived from the outer retina can be acted upon by electrical circuits in the inner retina to enhance correlated activity. Indeed, as shown for the broadest correlations, the fact that Cx36-expressing gap junctions are critical for their generation does not preclude the clear role for shared inputs.

### Role of Concerted Activity in Encoding Visual Information

Concerted activity is a common property of neuronal ensembles and its function in encoding and propagating information in the brain, including the visual system, appears multivariate. As noted, the robust concerted activity of retinal GCs may contribute to the enhancement of saliency or temporal precision of signals, compression of information for transmission through the optic nerve or temporal binding of visual information [11 15 24 30 51 52 53 54]. In contrast, some synchrony may reflect signal redundancy that may be irrelevant to information coding and decoding. It has been posited that reciprocal connections via gap junctions, as shown here to produce bimodal correlations may produce such redundancy, whereas shared inputs to GCs, either through common chemical synapses via bipolar cells or a common cohort of electrical coupled ACs could produce a more relevant distributed code [35 55]. The finding that the different forms of concerted activity have different generating mechanisms suggests that their function in encoding information also differs. For example, broad correlations produced by spatially segregated GCs can serve to encode certain features of the visual scene such as global objects important for perceptual grouping critical to object recognition [27]. More temporally precise concerted activity could control the spike timing of neighbors in which differences in latencies may be a mechanism for encoding visual information [56].

Our finding that the vast majority of light-independent GC correlations rely on electrical coupling in the inner retina and not primarily photoreceptor noise might imply that this mechanism is distinct from that used to encode visual signals. However, the fact that electrical coupling alone can produce concerted GC activity does not preclude its interactions with visual signals derived from the outer retina to modify the neural output code of the retina. Indeed, there is clear evidence for this. During daylight hours in which visual encoding must be maximized, the concerted firing of light-dependent spikes is increased due to enhanced electrical coupling in the inner retina [29]. It has recently been shown that indirect coupling of ON direction selective GCs via intermediary ACs induces concerted spiking that contribute to the coding of stimulus movement critical to the optokinetic response [13]. Thus, the same mechanisms responsible for light-independent correlated firing are evidently employed in creating the neural code in GC ensembles that maps the visual world.

## Materials and Methods

### Flattened Eyecup Preparation

Adult (P_30–90_) wild type (WT) and Cx36 knock-out (KO) mice with C57BL6/129SvEv hybrid background [57] were used in most experiments. In some experiments CB2-GFP mice generated on a Swiss-webster/C57BL6 hybrid background were used [41]. These mice expressed GFP controlled by the promoter for the calcium binding protein calretinin. Mice were deeply anesthetized with an intraperitoneal injection of Nembutal (0.08g/g body-weight) and lidocaine hydrochloride (20 mg/ml) applied locally to the eyelids and surrounding tissue. A flattened retinal-scleral preparation developed for rabbit [58] was adopted and modified for the mouse. Briefly, the eye was removed under dim red illumination and hemisected anterior to the ora-serrata. Anterior optics and the vitreous humor were removed and the resultant retina-eyecup was placed in a superfusion chamber. Several radial incisions were made peripherally allowing flattening the eyecup. The chamber was then mounted in a light-tight Faraday cage and superfused with an oxygenated mammalian Ringer solution (pH = 7.4, 32 °C [59]. Surgery was carried out under red illumination and retinas were dark adapted for 1 hour prior to experimentation. Animals were sacrificed immediately after enucleations. All animal procedures were in compliance with the NIH Guide for the Care and Use of Laboratory Animals and approved by the Institutional Animal Care and Use Committee at NYU School of Medicine.

### Visualization of Neurons

To visualize cells for recording in the ganglion cell layer a 890 nm cut-off filter allowed transmission of infrared (IR) light from below the stage and then up through a condenser and the glass coverslip mounted in the superfusion chamber. An IR sensitive CCD camera (Dage-MTI VE-1000) captured the retinal image that was displayed on a video monitor outside the Faraday cage. This paradigm allowed retinas to remain in the dark-adapted state during viewing. To visualize GFP expression in CB2 positive neurons in the living retina we used an epifluorescent mercury light source and a GFP filter.

### Electrophysiology

Extracellular recordings were obtained from neurons using either tungsten microelectrodes (Microprobe Inc. Gaithensburg, MD, USA) attached to an isolated AC differential amplifier (DAM80i, World Precision Instruments) or a MEA system (Multichannel Systems Gmbh, Germany) that allowed for recording simultaneously from up to 60 retinal cells. For pharmacological experiments, drugs (Sigma, St Louis, MO, USA) were applied to the retina by switching from the control Ringer solution to one containing either a cocktail of 50 µM MK-801, 10 µM CNQX, 50 µM PTX, 5 µM STR and 50 µM APB or the gap junction blocker 18-beta-glycyrrhetinic acid (18β-GA) in a concentration of 25 µM. All recorded data were digitized online with an analog-to-digital board (Digidata 1200; Axon Instruments, Sunnyvale, CA, USA) and stored for off-line analyses. Spike trains were recorded digitally at a sampling rate of 20 kHz with Axoscope (Axon Instruments, Foster City, CA) or MC Rack (Multichannel Systems Gmbh, Germany). For additional off-line analysis, Off-line Sorter (Plexon, Dallas, TX) and Neuroexplorer (Nex Technologies, Littleton, MA) softwares were used. Gaussian functions were fitted on cross-correlation functions with Origin (Microcal, Northampton, MA, USA) and the Neuroexplorer (Nex Technologies, Littleton, MA) softwares were used. The cross-correlation curves were fit by the Gaussian functions with Origin software (Microcal, Northampton, MA, USA) as:
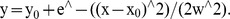
where *A* is amplitude and *w* is width.

In experiments in which pairwise recordings from GCs of the same subtype were performed, visualized somata of the same size and shape were targeted with tungsten electrodes. Following extracellular recordings, one cell of the pair was penetrated with an intracellular glass microelectrode under visual guidance for intracellular recording and/or labeling with Neurobiotin. Comparisons were then made of the mosaic of cell bodies in the live retina with that in histologically processed tissue to confirm that the recorded GCs were tracer coupled and to identify them as of the same morphological subtype. The microelectrodes were filled at their tips with 4% *N*-(2-amino-ethyl)-biotinamide hydrochloride (Neurobiotin, Vector Laboratories; Burlingame, CA, USA) in 0.1 M Tris buffer, pH7.4, and then back-filled with 4 M KCl. Final DC resistances of these electrodes ranged from 200 to 300MΩ. Neurobiotin was injected into the cell with a combination of sinusoidal (4 Hz; 0.4 nA; peak-to-peak) and direct current (0.4 nA) applied simultaneously. This method allowed for passage of tracer through the microelectrode without polarization.

### Light Stimulation

A green (λ = 525 nm) light-emitting diode delivered uniform full-field visual stimuli on the surface of the retina. The intensity of the square wave light stimuli was calibrated with a portable radiometer/photometer (Ealing Electro-Optics, Holliston, MA) and expressed in terms of the time-averaged rate of photoisomerizations per rod per second (Rh* per rod/sec). Light intensities were calculated assuming an average rod density of 437,000 rods/mm2 [60] and quantum efficiency of 0.67 [61]. The intensity of the light stimuli varied from 10^−2^ to 10^4^ Rh* per rod/sec. While most recordings were carried out in dark-adapted conditions, we also recorded activity in light adapted retinas. In these experiments, retinal samples were adapted for one hour prior to recordings with a fullfield photopic (I = 3000 R*/rod/sec) background light generated by a green LED (λ = 525 nm).

### Histology and Immunocytochemistry

One hour after labeling the last cell in an experiment, the retina was fixed overnight in a cold (4°C) solution of 4% paraformaldehyde in 0.1 M phosphate buffer (pH = 7.3). Retinas were then washed in phosphate buffer and soaked in a solution of 0.18% hydrogen peroxide in methyl alcohol for one hour. This treatment completely abolished the endogenous peroxidase activity. Retinas were then washed in phosphate buffer and reacted with the Elite ABC kit (Vector Laboratories, Burlingame, CA) and 1% Triton X-100 in sodium phosphate-buffered saline (0.9% saline, pH = 7.6). Retinas were subsequently processed for peroxidase histochemistry using 3,3′-diaminobenzidine (DAB), dehydrated, and flat mounted for light microscopy.

Digital images of labeled neurons were captured by a cooled CCD camera (Spot 2, Diagnostic Instruments, Sterling Heights, MI) followed by software manipulation of brightness and contrast (Photoshop, Adobe Systems, San Jose, CA). To determine the level at which dendritic processes stratified in the IPL, we examined Neurobiotin-labeled cells in flat mount under a 100X oil-immersion lens. The borders of the IPL were determined by the location at which AC and GC bodies were defocused using Nomarski interference contrast optics.

Alternatively, Neurobiotin injections in some retinas were visualized using a Cy3-conjugated streptavidin reagent (Sigma). After labelling with streptavidin-Cy3, retinas were washed in PBS for 1 h. Subsequently, retinas were incubated in a primary antibody solution of mouse anti-connexin36 (Cx36) antibody (Chemicon) at a concentration of 1∶2000 or rabbit anti-GFP antibody (Molecular probes) at a concentration of 1∶1000. Retinas were then washed for 2 h in PBS, before incubation in a secondary antibody solution of donkey anti-mouse Cy2 (1∶500) or goat anti-rabbit Alexa 488 overnight at 4°C (Jackson ImmunoResearch Laboratories). Retinas were subsequently washed in PBS for 3 h and mounted in Vectashield mounting medium (Vector Laboratories). Retinas were imaged using a Zeiss 510Meta confocal microscope (Zeiss; Thornwood, NY, USA).

### Purification of GFP-Expressing Retinal Ganglion Cells

P5 mouse retinal GCs from BAC-transgenic lines expressing GFP in a defined subtype of GCs 41) were purified based on previously described immunopanning procedures [62] and positively selected using a monoclonal Thy1.2 antibody (Serotec, Cat# MCA02R). The acutely purified neurons were then resuspended in panning buffer containing 0.2% BSA and propidium iodide, and immediately sorted by FACS to select for viable GFP-expressing GCs. Cells were sorted twice to yield >99.5% purity based on reanalysis of double sorted cells.

### RNA Preparation, Microarray Hybridization, and Data Analysis

Total RNA from the small sample of fluorescence-activated cell sorted (FACS), GFP positive GCs (approx. 200–300 neurons) were extracted using a commercial kit (PicoPure RNA Isolation Kit, Applied Biosystems Cat#KIT0204), and the RNA concentration/quality were assessed with a NanoDrop spectrophotometer. Next, 2 rounds of amplification was performed to synthesize sufficient final cRNA content for GeneChip hybridization based on procedures modified from the original Affymetrix GeneChip Eukaryotic Small Sample Target Labeling Assay Version II protocol (www.Affymetrix.com). Full, modified protocol is available upon request. Briefly, total RNA volume was concentrated using a speed-vac down to 1 µl. First and second strand cDNA were then generated with stock T7-oligo-dT primers (5′-GGCCAGTGAATTGTAATACGACTCACTA TAGGGAGGCGG-(dT)24-3′) based on standard reverse transcription procedures. This newly synthesized cDNA was then precipitated in EtOH overnight in −20 degrees. Next, the 1^st^ round cRNA was synthesized by in vitro transcription (MEGAscript T7 Kit, Ambion Cat#1334) and purified (RNEasy kit, Qiagen Cat#74104) using commercially available kits. The cRNA was then reverse transcribed again to generate 2^nd^ round cDNA using the same reverse transcription procedures as before. This amplified cDNA is further transcribed into biotin-labeled cRNA using an RNA transcript labeling kit (High Yield Bioarray RNA transcript labeling Kit, Enzo Cat#42655-10). 20 µg of labeled cRNA was then fragmented using appropriate fragmentation buffer in 94°C for 30 min and hybridized onto a mouse genome 430 2.0 array per Affymetrix instructions (www.Affymetrix.com) to probe against over 39,000 transcripts.

Raw image files were processed using Affymetrix GCOS and the Microarray Suite (MAS) 5.0 algorithm. Intensity data were normalized per chip to a target intensity value of 500 to obtain standardized expression data across all individual probe sets. Expression threshold for filtering present vs. absent transcripts was set at MAS 5.0 intensity level of 200, in which transcript expression values above 200 were deemed present and those below were absent. Transcript expression values were compared amongst GCs in specific GFP-expressing BAC transgenic lines as well as to global GC transcripts.

To evaluate Cx36 mRNA expression in GFP positive GCs and in all retinal GCs we compared arbitrary values to those obtained for either negative controls (transcripts not expressed in GCs) or with positive controls (expressed in all GCs). We chose Cx57 as a negative control because it is expressed in the retina but not by GCs [63 64 65 66], Cx32 and Cx37, two connexins with no known retinal expression, and tyrosine-hydroxylase (TH) that is expressed in retinal ACs but not in GCs. We chose the GABA_R_-α2 subunit mRNA as a positive control because it is ubiquitously expressed in the inner retina.

### Statistics

Student’s *t*-test was used to evaluate statistical significance.
